# Ecological Characterisation of Native Isolates of *Heterorhabditis indica* from Viti Levu, Fiji Islands

**DOI:** 10.21307/jofnem-2021-085

**Published:** 2021-10-28

**Authors:** Sumeet Kour, Uma Khurma, Gilianne Brodie

**Affiliations:** 1 School of Agricultural, Geography, Environment Ocean and Natural Science, The University of the South Pacific, Fiji Islands; 2 Institute of Applied Sciences, The University of the South Pacific, Fiji Islands

**Keywords:** Biology, Ecological characterisation, Fiji, Entomopathogenic nematodes

## Abstract

Entomopathogenic nematodes (EPNs) in the families Steinernematidae and Heterorhabditidae are obligate parasites of soil inhibiting insects. EPNs are being widely researched as promising biocontrol agents for a wide range of agricultural pests. It is known that strains of EPNs isolated from different geographical regions differ in their attributes, such as host-finding ability, host range, infectivity, reproduction, and environmental stress tolerance. A precise knowledge of these factors is therefore an essential pre-requisite for devising successful strategies to use these nematodes in biological control programmes. Thus, ecological characterisation of the EPN *Heterorhabditis indica* (Rhabditida: Heterorhabditidae) newly isolated and representing the only species of EPN reported from the island of Viti Levu, Fiji was carried out using *Galleria mellonella* larvae (L) (Pyralidae: Galleriinae) as hosts to allow comparisons between bioassays conducted in different laboratories around the world. Temperature data showed that native isolates of *H. indica* are warm-adapted nematodes with thermal range for infectivity between 15˚C and 35˚C and can reproduce between 20˚C and 30˚C. They are highly virulent with LC50 values against *G. mellonella* ranging from 2.8 IJ to 3.8 IJ/larva. However, they showed poor desiccation tolerance and fail to infect hosts in soil with moisture levels below 8%. They showed a moderate level of hypoxic tolerance and can be stored at 15˚C for 4 months. Results also showed great variability within the selected native isolates of *H. indica.* Beneficial traits for selected isolates were added up to identify a superior candidate. The current study also suggested that the thermal niche breadth for infection can differ among conspecific strains of an EPN species. The results of this experimental study on ecological aspects of these native isolates of *H. indica* should form a basis for their potential use in biological control of insect pests in Fiji.

Entomopathogenic nematodes (EPNs) species belonging to the genera *Steinernema* (Travassos, 1927) and *Heterorhabditis* (Poinar, 1975) and their symbiotic bacteria from genera *Xenorhabdus* and *Photorhabdus*, respectively, are obligate parasites of soil inhibiting insects (Shapiro-Ilan et al., 2017). Because of the increasing awareness of EPN as an effective non-chemical alternative to control insect pests, many surveys have been and are being carried out across the globe to isolate new species and/or population of these nematodes that are either more virulent and/or locally adapted to the region’s environmental conditions. According to Seenivasan and Sivakumar (2014), native EPN can be commercially viable if they possess high virulence against targeted pest, high tolerance to environmental stress (hence enhanced survival and persistence), high reproductive capacity for mass production, longevity and increased storage life when commercially formulated. Hence, for successful use of native EPNs as biocontrol agents, a knowledge of their ecology is of paramount importance (Salame et al., 2010). Since, EPNs are extracted from soil samples using insect baits, only a few species have been isolated from natural insect hosts; our understanding, of their biology and ecology is very limited (Nguyen and Buss, 2011; Pilz et al., 2008). Many of these isolates currently being cultured in laboratories around the world remain ecologically undescribed. Taxonomic identification of new species or strains or isolates should be followed by detailed study of their ecological traits in order to optimize their biocontrol potential (Koppenhöfer and Kaya, 1999) as they may have different ecological traits that could make them potentially more useful than those currently in use (Vinciguerra and Clausi, 2006). Superior traits can increase field efficacy of EPN, thus reducing the total overall cost for controlling the pests (Ma et al., 2013).

Ecological characterisation of *H. indica* isolated from different geographical regions have been reported previously (Ebssa et al., 2004; Iraki et al., 2000; Karunakar et al., 1999; Ma et al., 2013; Nguyen et al., 2006; Noosidum et al., 2010; Shamseldean et al., 1996; Shapiro-Ilan et al., 2005; Shapiro-Ilan et al., 2014; Shapiro-Ilan et al., 2009; Walia et al., 2008; Xu et al., 2010; Yadav, 2012). In the present study, we provide the ecology of the EPN *H. indica* isolated from the island of Viti Levu, Fiji. The native isolates of *H. indica* in this study have been isolated from different climatic zones and diverse habitats (Kour et al., 2020). The isolated population of *H. indica* have shown some degree of morphometric and genetic variability (Kour et al., 2020). Therefore, difference in activities or behaviour would be expected among these isolates. Thus, ecological characterisation of native populations of *H. indica* was carried out to provide basic knowledge to develop native isolates of *H. indica* as a biological control agent for native insect pests listed in The Pacific Islands Pest List Database (https://www.spc.int/pld/). Many of these economically important agricultural insect pests listed in database have been targeted in other countries using EPNs with satisfactory results. In this study, the native isolates were characterised by determining their host finding ability, the effect of temperature on their infectivity and reproduction, the effect of soil moisture on their infectivity, heat tolerance, desiccation tolerance, hypoxia tolerance, and effect of storage temperature and duration on their survival. A precise knowledge of these factors is an essential pre-requisite for devising successful strategies to use native isolates of *H*. *indica* as biological control agents.

## Materials and methods

Koppenhöfer and Kaya (1999) outlined simple experiments that can serve as model for ecological studies. Their recommendations on standard ecological experiments were followed primarily for bioassays of the native isolates of *H. indica* in this study. Suggestions made by other researchers such as Shapiro-Ilan et al. (2005), Gungor et al. (2006), Salame et al. (2010), Ma et al. (2013), and Zadji et al. (2014) were incorporated as applicable.

### Nematodes, host insect, and soil

Culture of all the 35 isolates obtained during the survey of Viti Levu was maintained separately under laboratory conditions. Keeping isolates separately prevented their contamination with other isolates and preserved their genetic diversity (Kaya and Gaugler, 1993). All isolates were reared in last-instar of the host insect, *G. mellonella* at 25°C. IJ were harvested three to 5 days after their first emergence from the cadaver and were stored at 15°C as aqueous suspensions (in distilled water) in 750 ml tissue culture flasks for no longer than 6 days. Prior to testing, IJ were washed with distilled water and their viability was determined by microscopic examination (Agudelo-Silva et al., 1987). The viability was determined by counting the live nematodes under the microscope. If sample has more than 95% live nematode it was used in experiment. The concentration of IJ required in each bioassay was estimated from the mean of ten subsamples (1 ml) from each nematode suspension and final concentration was adjusted by volumetric dilutions. Since the extended laboratory culture of EPNs in the absence of outbreeding can be deleterious (Kaya and Gaugler, 1993), the strains used were kept in culture for less than five generations (Selvan et al., 1994; Shapiro et al., 1996). Also, to ensure production of IJ with good biological activity and virulence, the population was maintained by trapping the first emerging IJ and using them for initiating the next generation of IJ (Lindegren et al., 1993). The last instar of *G. mellonella* with average weight of 0.2 g to 0.3 g was used as test insect. *G. mellonella* is universally preferred experimental host insect for EPNs. Hence, its use allows comparisons between bioassays conducted in different laboratories around the world (Grewal et al., 1999). Except for single dose screening assays, use of soil was preferred over tissue paper in infectivity assays. Hence, soil with sandy loamy texture (73% sand, 10% silt, 17% clay) with 1.3% organic matter was used in bioassays.

### Single-dose screening assay

A single-dose screening assays was conducted for all 35 nematode isolates to test their relative virulence against *G. mellonella* and to select the three top ranked isolates for further ecological characterisation. For single-dose screening assay, a 5.5-cm Petri dish was lined with a moistened filter paper. Approximately 50 IJ were transferred to each Petri dish to which five *Galleria* larvae were added. The host mortality and time duration to death were recorded. Based on mean time until death and mean proportional mortality, the three most virulent isolates were selected for further ecological characterisation.

### Foraging strategies and host finding ability

Three experiments were conducted to determine the type of foraging and host finding strategy used by native isolates of *H. indica*. In the first experiment, a 100-mm Petri dish was lined with a moistened filter paper and sprinkled with 0.2 g of sand particles. Approximately, 100 IJ were transferred to each Petri dish, and the behaviour of the IJ was monitored for 10 min using a dissecting microscope at × 50 (Gungor et al., 2006). There were five replicates per isolate. A second experiment was conducted to evaluate the response of IJ to the host volatile cues. The experiment was conducted as described by Shapiro-Ilan et al. (2005). A 100-mm Petri dish was filled with 2% agar. Two 1000 µl pipette tips were inserted in the lid of Petri dish on the opposite side to each other. On a treatment plate, a *G. mellonella* larva was placed in one pipette tip and the other tip was left empty. In control plates, both tips were left empty. The Petri dish was sealed with paraffin film and was left for 1 hr to build the host cue gradient. After an hour, approximately 2000 IJ were placed on a small piece of filter paper positioned in the centre of the agar plate through a hole in the cover. After 2 hr, the number of IJ found in a 1 cm diameter circle under each pipette tip was recorded. Host-seeking ability was estimated as percentage of IJ found under the pipette tip with the host relative to the total number of IJ found under the host plus those found under the empty pipette tip. In control plates, none of the pipette tip had insect host and one side was randomly designated the host side for calculation purposes. There were five replicates for each isolate. In the third experiment, the ability of the IJ to find and infect a sedentary host at different soil depths was determined (Raja et al., 2011). The soil column bioassay was used to study the ability of the IJ to find hosts and calculating the invasion efficiency at different depths. A plastic tube of 11.5 cm height was filled with sandy loam soil up to 10.5 cm. A *G. mellonella* larva was constrained between the two layers of aluminium mesh that did not allow the larva to crawl. The aluminium mesh was placed at 0, 2, 5, or 10 cm soil depth, and 100 IJ in 1 ml of distilled water were added on the surface of each column. There were five replicates per isolate. After 2 days the columns were disassembled and the dead larvae were dissected and pepsin-digested to determine the number of nematodes that had penetrated into the body cavity of the host (Koppenhöfer and Fuzy, 2003). For pepsin digestion, the dissected cadaver was placed in 15-ml centrifuge tubes containing 4 ml of pepsin solution (2.3 g of NaCl and 0.8 g of pepsin in 100 ml of deionized H_2_O, adjusted to pH 1.8 to 2.0 with conc. HCl). The tubes were incubated in water bath at 37°C for 1½ hr. Following incubation, 10 ml of 0.1% Tween80 solution was added to each tube. The tubes were vortexed for 20 sec and contents were poured into a 10-cm Petri dish. All nematode stages present in the suspension were counted under × 50 magnification.

### Ability to kill insects (LC50)

LC50 of three isolates of *H. indica* were investigated against *G. mellonella* larvae. A 50 µl drop containing 1, 3, 5, 10, and 25 IJ were applied to 24-well tissue culture plate. To control plate only 50 µl of distilled water was added. Then two grams of sandy loamy soil, the moisture of which was adjusted to 12% (w/w), was added to each well. One last-instar of *G. mellonella* was added to each well. The plates were covered and incubated at 25°C. Mortality was assessed after 48 hr by probing. In the absence of a response to the probe, the larva was considered dead. Twenty *G. mellonella* larvae were tested per concentration.

### Pathogenicity at different temperatures

The experiment was conducted to evaluate pathogenicity of the three selected native isolates of *H. indica* at different temperatures using host *G*. *mellonella* and to establish the range of temperatures at which the native isolates can infect insect larvae. Initially, the experiment was conducted as described by Koppenhöfer and Kaya (1999) but in the second attempt slight changes were made in methodology. In the first failed attempt, air dried soil was used, whereas in second attempt a moisture level of the soil was adjusted to 12% (w/w). Three 24-well tissue culture plates were used. 50 IJ in 20 µl of distilled water were applied to each well. The plates were placed in incubators at 10^°^C, 15^°^C, 20^°^C, 25^°^C, 30^°^C, 35^°^C, and 40^°^C. After 1 hr for acclimatisation, one last instar larva of *Galleria* was added to each well. The plates were covered in polythene bags to avoid evaporation and desiccation of IJ. The first plate was used to record larval mortality, time of death, and number of nematodes that penetrated per larva at various temperatures. To record time of death, the wells were examined at 12 hr interval for 3 weeks and each larva was probed. In the absence of response to the probe, the larva was considered dead. Dead larvae were removed from the wells and thoroughly washed in distilled water. The cadavers were dissected and digested in a pepsin solution (Mauleon et al., 1993). Dissection and digestion were done 1 day after death at 25°C and 35°C, 2 days after death at 20°C, and 3 days after death at 15°C. The second plate was used to record the time of the first emergence of progeny at various temperatures. The plates were checked daily and cadavers were removed from the plate and placed in a Petri dish and further incubated at respective temperatures until the emergence of the IJ. The White traps were monitored daily and the first day of IJ emergence was recorded. Cadavers from which no IJ had emerged were dissected to determine the fate of the infection. The third plate served as a control to which only 20 µl of distilled water was added.

### Reproductive capacity at different temperatures

Experiments were conducted to evaluate the reproductive capacity of three selected native isolates of *H. indica* in host *G*. *mellonella* at different temperature and to establish reproduction thermal niche breadth of these isolates. Each well of 24-well tissue culture plates was filled with 0.5 g of sterile, air dried sandy loamy soil whose moisture level was adjusted to 12% (w/w). To each well 50 IJ in 20 µl of distilled water were applied. One last instar larva of *Galleria* was added to each well. The plates were kept at 25°C for 1 day to standardise the number of IJ penetrating into the larvae. The plates were placed in incubators at 10°C, 15°C, 20°C, 25°C, 30°C, 35°C, and 40°C and were covered in polythene bags to avoid evaporation and desiccation of IJ. The cadavers were removed from the tissue culture plate, thoroughly washed to remove soil or any nematode attached on the surface and placed in White traps which were further incubated at respective temperatures until the emergence of the IJ. Small White traps were used for easy collection of all the emerging IJ. The Small White traps were made using 5.5-cm Petri dishes and small plastic lids. The plastic lid was inverted and placed in Petri dish. It was lined with filter paper with edges touching the water in the outer Petri dish. There were 24 cadavers per isolate per temperature. The White traps were monitored daily. The IJ were harvested till they stop emerging and pooled together and stored at 15^°^C. Ten White traps were randomly selected for counting the total number of IJ emerging from the cadaver and progeny was estimated by counting five subsamples under ×50 magnification.

### Thermal tolerance

Before the experiment, the IJ stored at 15°C were allowed to adapt to room temperature (25°C) for 2 hr. Thermal tolerance was evaluated as described by Ma et al. (2013) with a little modification. Approximately 2000 IJ were suspended in 1 ml of Ringer solution (9 g NaCl, 0.4 g KCl, 0.4 g CaCl_2_ and 0.4 g Na_2_CO_3_ dissolved in 1000 ml of distilled water and autoclaved) (Lacey, 1997) and incubated at 40°C for 1, 2, 3, and 4 hr. After each interval period, 9 ml distilled water was added to the tube and tubes were kept for an additional 24 hr at room temperature (25°C) to allow the nematodes to recover from the heat shock. A sample of 50 µl was taken for recording the live and dead nematodes. Live nematodes were counted using a stereomicroscope. Nematodes were considered live if they were naturally moving or responded to probing with a fine needle. For each isolate, IJ maintained at 25°C were used as control. There were five replicates for each isolate.

### Infectivity at different soil moisture level

An experiment was conducted to evaluate the effect of soil moisture on the capability of seeking out and infecting host *G*. *mellonella* and to establish the range of moisture levels at which they can infect host larvae. 100 IJ in 20 μl of water were placed on the bottom of each well of 24-well tissue culture plates. The wells were filled with 2 g of sterile, air dried sandy loamy soil whose moisture level had been adjusted to 5%, 8%, 10%, 15%, 20%, and 25% (w/w). One last instar larva of *Galleria* was added to each well and wells were completely filled with soil. In control plate only 20 µl of distilled water was added. The plates were incubated at 25°C, covered in polythene bags to avoid evaporation and desiccation of IJ. The dead larvae were recovered after 3 days and washed thoroughly to remove soil or any nematode attached on the surface. The cadavers were dissected and enzymatically digested to record the number of nematodes that had penetrated into it. Since it was not possible to dissect all cadavers at the same time, the cadavers were stored in the freezer to be dissected later. There were twenty-four replicates per soil moisture level.

### Rapid desiccation tolerance

Desiccation tolerance of native isolates was evaluated based on the procedure described by Shapiro-Ilan et al. (2005). Approximately 2000 IJ of each isolate were applied to 5-cm diameter filter paper disks (Whatmann No. 1). The excess water was removed from the filter paper disc by vacuum filtration. The filter paper containing IJ was placed on a lid of a 5-cm Petri dish and left open at room temperature (25°C) for 15 min for excess moisture to dry. Later, they were transferred to desiccator and exposed to relative humidity (RH) levels of 85%. RH level of 85% was established by using 60 ml of saturated salt solutions of KCl (Solomon et al., 1999). After 24 and 48 hr, the IJ were rehydrated by immersion in 10 ml of sterile water for another period of 24 hr. The number of dead and live IJ was counted in 50 µl of sample using a stereomicroscope. The IJ were considered dead if they did not show any mobility and response on probing with a needle. All treatments were incubated at 25°C. There were five replicates for each isolate.

### Hypoxia tolerance

Hypoxia tolerance was evaluated as described by Zadji et al. (2014) with slight modification. Approximately 10,000 IJ were suspended in 0.5 ml distilled water and transferred to a 0.5 ml Eppendorf tube. At this concentration, the dissolved oxygen level can reach zero within 10 min (Grewal et al., 2002). The tubes were closed and kept in the dark at 25°C for 24 hr and 72 hr. After treatment, the nematodes were suspended into 15 ml water in a Petri dish and kept at 25°C in the dark for an additional 24 hr. Open Eppendorf tubes constantly kept at 25°C in the dark were used as controls. 50 µl of sample was taken for recording the live and dead nematodes in the sample and counting was done using a stereomicroscope. There were five replicates for each isolate.

### Survival capacity in storage

To determine the optimal storage conditions for *H. indica*, IJ were collected and diluted to 1000 IJ/ml with sterilized deionized water. A concentration of 1000 IJ/ml is the best to store Heterorhabditidae (Acevedo et al., 2006). 50 ml of this suspension was collected in BD falcon 750-ml tissue culture flask and stored at 5°C, 10°C, 15°C, 20°C, and 25°C. There were two replicate flasks for each temperature and flasks were sampled after 1, 2, 4, 8, and 16 weeks. Survival was determined in subsamples of approximately 100 IJ per flask under a dissecting microscope. Nematodes were considered live if they were naturally moving or responded to probing with a fine needle.

### Qualitative analysis of ecological traits among isolates

To facilitate comparison among isolates, the performance of each isolate for each ecological trait was scored as described by Salame et al. (2010). The isolate was scored as 1 if performance was not significantly different from the highest level for that trait, -1 if performance was not significantly different from the lowest level for that trait, and 0 if performance was between the highest and lowest, or not significantly different from either. The scores among traits were then added for each isolate and comparison was made.

### Data analysis

Statistical analyses were performed on the data collected from each set of experiments. Excluding the single-dose screening assays and survival capacity in storage, all the experiments were conducted twice. To investigate the difference between the two trials independent samples t-test was used. In all cases, the results of both trials were similar and were combined for analysis. For screening test, the time until death and proportional mortality were calculated, and isolates were ranked. Isolates having low rank for time until death and proportional death were selected. For rest of the experiments, before conducting statistical analysis all percentage data was transformed using the arcsine square root transformation. For reproduction assay, the data was normalized by log transformation (Campbell and Wraight, 2007). Non-transformed means are presented in figures. For host cue experiment, Independent samples t-test was conducted between each isolate and corresponding control to study the response of individual isolate toward the host volatile cues. When required, insect mortality data was corrected according to Abbott (1925). For vertical soil experiment data was assessed using analysis of variance (oneway ANOVA) at the 5% level of significance (Freund and Littell, 1981) and Tukey’s test was used to compare means. LC50 values of EPN isolates were calculated using probit analysis (Finney, 1971). Overlap of the 95% fiducial limits was used to determine significance (Taylor et al., 1998) and values were not considered to be significant whenever the ranges overlapped (Grewal et al., 1993). The effect of storage temperature on survival was analysed using repeated measures analysis of variance (Koppenhöfer and Kaya, 1999). Statistical analysis was performed using SPSS 21.0 software for Windows XP.

## Results

### Single-dose screening assay

**Figure 1:**
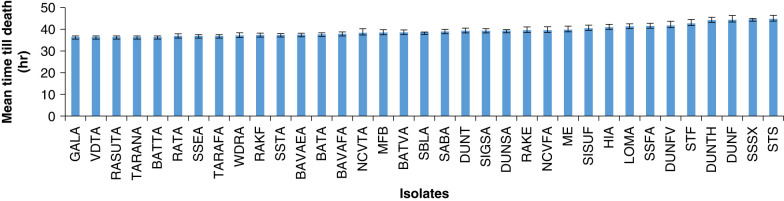
Time till death in hours (mean ± SE) of host *Galleria* caused by *H. indica* isolates from Viti Levu.

**Figure 2:**
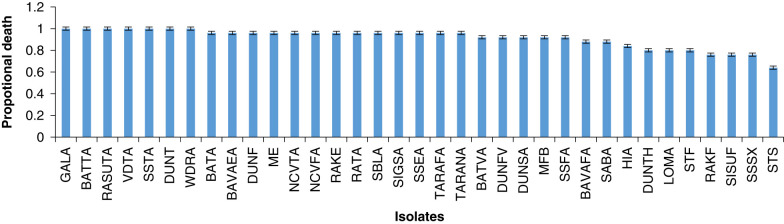
Proportional death (mean ± SE) of host *Galleria* caused by *H. indica* isolates from Viti Levu.

The time taken by different isolates to kill the insect host ranged from 36.48 ± 1.07 hr to 44.8 ± 3.7 hr. Isolates GALA, VDTA, RASUTA and BATTA took least time (36.48 ± 1.07 hr) to cause 100% mortality, whereas, isolate STS took maximum time (44.8 ± 3.7 hr) to cause 64% of host mortality. The 35 isolates were ranked according to the time until death and proportional mortality (Figs. 1, 2).

Two isolates, GALA and VDTA were selected as both showed 100% host mortality in least time (36.48 ± 1.07 hr) and they both belong to different climatic regions of Viti Levu. DUNT also recorded 100% mortality but took more time to cause mortality (39.3 ± 2.7 hr). DUNT was selected for further ecological characterisation because it was suspected to harbour a different species of symbiotic bacteria. Incidentally, all the three isolates were recovered from different locations as well as different habitat type. Isolate GALA was recovered from Galoa (Windward side of the island) along riverside among wild ginger plants. Isolate VDTA was recovered from grass land in Vuda region (Leeward side of the island) and Isolate DUNT was recovered from sand dune along coastline in Sigatoka.

### Foraging behaviour of *H. indica*

**Figure 3:**
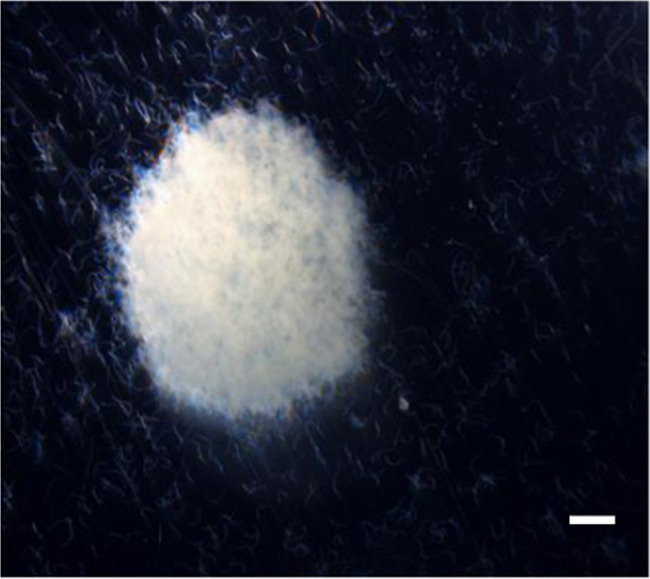
Dense aggregate of *H. indica* IJ in aqueous suspension. Bar = 1 mm.

All the IJ of native isolates of *H. indica* were seen moving on the surface and did not show any standing, body waving or jumping behaviour. However, in storage, in aqueous suspension, dense aggregation of IJ was observed (Fig. 3).

**Figure 4:**
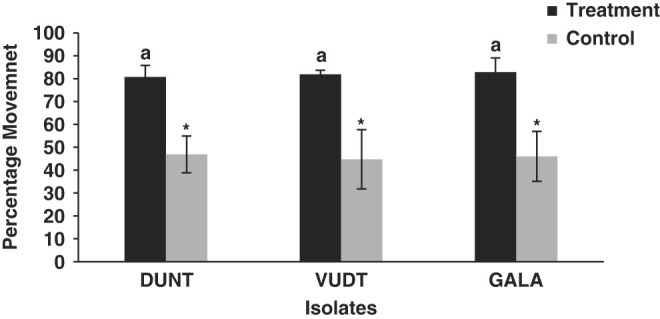
Mean percentage of IJ of isolate DUNT, VDTA and GALA that moved on agar-filled petri dishes toward *G. mellonella* host cues (treatment), or toward side of a dish that did not contain any host cues (control). The same lowercase letter above bars indicate no statistical differences among nematode isolates (*P* > 0.05); asterisks indicate significant differences between Treatment and Control dishes within each isolate (*P* < 0.05).

All the three isolates were highly attracted towards the volatile cues given by host insect and showed positive movement toward the host (Fig. 4). There were significant differences in percentage movement of IJ between treatment and control for three isolates for isolate GALA, treatment plates (Mean percentage ‘*M*’ = 82.8, Standard Deviation ‘SD’ = 6.2) and control (*M* = 46, SD = 10.9); *t* = -6.4, df = 8, *p* = 0.002; for isolate VDTA treatment plates (*M* = 81.8, SD = 1.7) and control (*M* = 44.7, SD = 12.9); *t* = -6.3, df = 8, *p* < 0.001 and for isolate DUNT treatment plates (*M* = 80.7, SD = 5.02) and control (*M* = 46.9, SD = 8.06); *t* = -7.8, df = 8, *p* < 0.001. However, there was no difference in percentage movement of IJ among three isolates of *H. indica* (*F* = 0.28; df = 2; *P* = 0.75).

**Figure 5:**
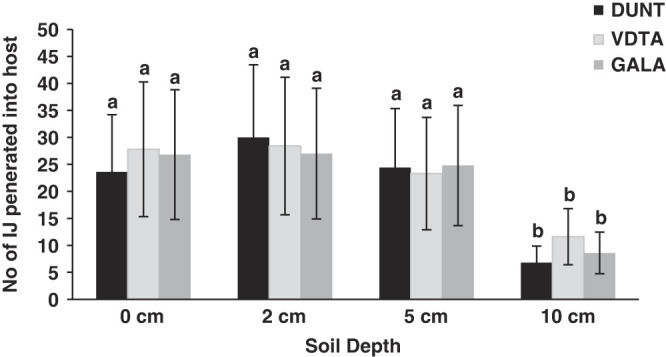
Effect on depth of host in soil (mean ± SE) on penetration of IJ of isolate DUNT, VDTA and GALA in *Galleria* larvae. Individual larvae were placed at 0, 2, 5, and 10 cm depth in soil and soil surface was treated with 100 IJ. Same lowercase letter above bars indicate no statistical differences among nematode isolates (*P* > 0.05).

In the vertical soil columns, all the isolates were able to reach to the depth of 10 cm and caused 100% mortality of host. The number of nematodes that penetrated into host at different soil depth was highest at 2 cm depth and lowest for 10 cm depth. At 2 cm depth, 30 ± 5.3 IJ/larvae, 28.4 ± 4.7 IJ/larvae and 27 ± 2.7 IJ/larvae were recorded for isolate DUNT, VDTA and GALA, respectively (Fig. 5), whereas, at 10 cm depth, 6.8 ± 1.7 IJ/larvae, 11.6 ± 1.7 IJ/larvae and 8.6 ± 3.2 IJ/larvae were recorded for isolates DUNT, VDTA and GALA, respectively (Fig. 5). There was no significant difference in penetration rate at 0 cm, 2 cm or 5 cm (*P* > 0.05) for all the tested isolates. However, at 10 cm all the three isolates showed significantly less penetration rate than the other depths (*F* = 70.5; df = 3; *P* < 0.001). There were no significant differences in penetration rates among three tested isolates of *H. indica* (*F* = 0.9; df = 2; *P* = 0.38) at different soil depth.

### LC 50 bioassays

The LC50 values of three EPN isolates DUNT, VDTA and GALA against *G. mellonella* ranged from 2.8 IJ/larvae to 3.8 IJ/larva in bioassays at 48 hr after application (Table 1). The dose-response test (LC50) revealed no significant differences among three tested isolates of *H. indica* χ2 (11, *N* = 3) = 2.06, *P* = 0.99.

**Table 1.** LC50 of native isolates of *H. indica* DUNT, VUDT AND GALA against last instar of *G. mellonella* larvae at 48 hr after application.

% Mortality^a^							
Concentrations (IJ/larva)							
Isolate	1	3	5	10	25	LC 50 (IJ/larva)	95% confidence interval
Isolate DUNT	15Aa	45Bb	75Cc	100Dd	100Dd	3.3	2.5-4.1
Isolate VDTA	10Aa	60Bb	85Cc	100Dd	100Dd	2.9	2.1-3.6
Isolate GALA	20Aa	55Bb	80Cc	100Dd	100Dd	2.8	2.1-3.6

Notes: ^a^Means followed by the same letters are not significantly different at the 5% level as determined by Tukey test (*a* = 0.05). Capital letters compare means in columns; small letters compare means in rows.

### Pathogenicity at different temperature

**Figure 6:**
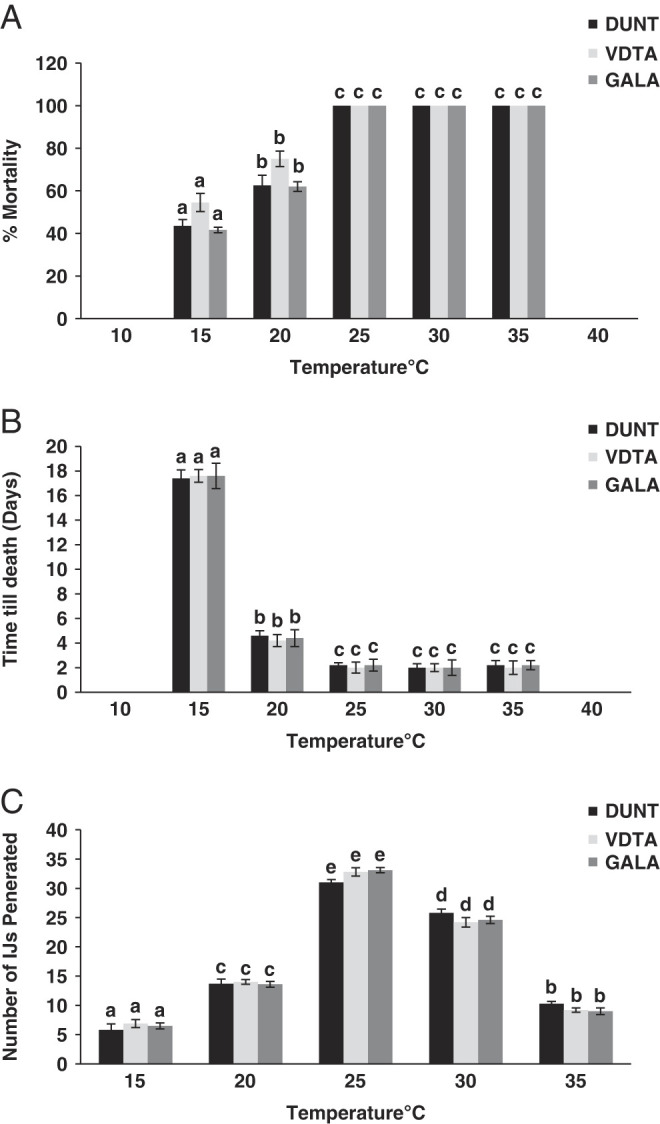
Pathogenicity of native isolates of *H*. indica at different temperatures. (A) Percentage mortality (Mean ± SE) of Galleria larvae when treated with IJ of isolat DUNT, VDTA and GALA at 10°C, 15°C, 20°C, 25°C, 30°C, 35°C, and 40°C. No *Galleria* larval mortality was observed at 10°C and 40°C. (B) Time to death (Mean ± SE) of *Galleria* larvae when treated with IJ of isolate DUNT, VDTA and GALA at 10°C, 15°C, 20°C, 25°C, 30°C, 35°C, and 40°C. (C) Number of IJ penetrated (Mean ± SE) into *Galleria* larvae when treated with IJ of isolate DUNT, VDTA and GALA at 15°C, 20°C, 25°C, 30°C, and 35°C. Same lowercase letter above bars indicate no statistical differences among nematode isolates (*P* > 0.05).

All the tested isolates of *H. indica* were able to infect and cause mortality of *Galleria* larvae at temperature range of 15°C to 35°C (Fig. 6A). No host mortality was observed at 10°C. At 40°C, larval mortality in treatment and control were equal and no penetrated nematodes were observed in treated larva. The highest mortality occurred between 25°C and 35°C (100% for all the three isolates), somewhat less at 20°C (62% for isolate DUNT and GALA; 75% for isolate VDTA); and low mortality at 15°C (45% for isolate DUNT; 58% for isolate VDTA; 41% for isolate GALA) (Fig. 6A). Temperature had a significant effect on the time of death (*F* = 3603.6; df = 4; *P* < 0.001). Time to death of larvae was shortest at temperature ranging from 25°C to 35°C (2-2.2 days) and longest at 15°C (17.4-17.7 days) (Fig. 6B). There was no significant difference in time to death at temperature 25°C, 30°C, and 35°C. Time taken by three isolates to kill the host at different temperatures was also not significantly different (*F* =1.9; df = 2; *P* =0.14). Number of nematodes penetrating into host larvae was highest at 25°C (31 ± 2.9 IJ/larva for isolate DUNT; 32.8 ± 3 isolate VDTA: 33.1 ± 3.4 for isolate GALA) and the lowest was observed at 15°C (5.8 ± 1.9 IJ/larva for isolate DUNT; 6.9 ± 2.3 isolate VDTA; 6.5 ± 2.4 for isolate GALA) (Fig. 6C). There were significant differences in number of nematodes that penetrated host at different temperatures (*F* = 378.6; df = 4; *P* = < 0.001). However, there was no significant difference in penetration rate among the isolates (*F* = 0.4; df = 2; *P* = 0.9) at different temperatures.

### Reproductive capacity at different temperature

**Figure 7:**
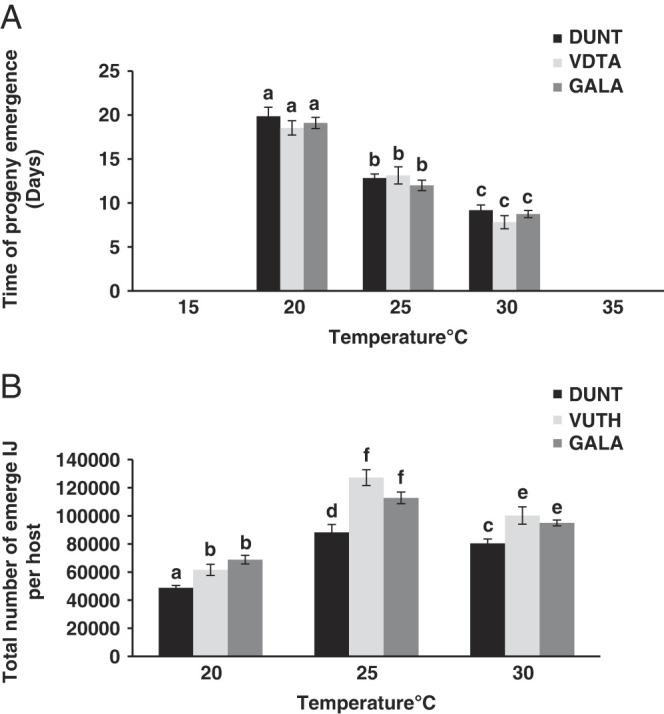
Reproductive capacity of native isolates of *H. indica* at different temperature. (A) Time of first progeny emergence (mean ± SE) of IJ of isolate DUNT, VDTA and GALA at 20°C, 25°C, and 30°C from *Galleria* cadavers. No IJ emerged at 15°C and 35°C. (B) Total number (mean ± SE) of emerged IJ of native isolates DUNT, VDTA and GALA at 20°C, 25°C, and 30°C from *Galleria* cadavers. Same lowercase letter above bars indicate no statistical differences among nematode isolates (*P* > 0.05).

Progeny emergence from cadavers was observed between 20°C and 30°C with no IJ emerging at 15°C and 35°C (Fig. 7A). Although insect larval death occurred and the nematodes established in the cadavers, no IJ emerged at 15°C and 35°C. At 20°C, only 37%, 50%, and 45% of the cadavers of isolate DUNT, isolate VDTA and isolate GALA respectively, produced progeny. At 25°C to 30°C, 100% cadavers for all the three isolates produced progeny. The shortest emergence time of IJ was observed at 30°C (7.8 ± 1.51 days) for isolate VDTA and the longest was at 20°C (19.7 ± 1.87 days) for isolate DUNT (Fig. 7A).

There were significant differences in day of emergence at different temperatures (*F* = 261; df = 2; *P* < 0.001). However, no differences in day of emergence was detected among three tested isolates of *H. indica* (*F* = 1.9; df = 2; *P* = 0.14) at different temperatures. The highest number of emerged IJ was observed at 25°C (average 127,000 for isolate VDTA, 112,800 for isolate GALA, and 88,200 for isolate DUNT) and the lowest number was observed at 20°C (average 61,600 for isolate VDTA, 68,800 for isolate GALA, and 48,800 for isolate DUNT) (Fig. 7B). The number of emerged IJ at 25°C was significantly higher (*F* = 168.8; df = 2; *P* < 0.001) than at 20°C and 30°C. Isolate DUNT progeny production was significantly lower than isolate VDTA and isolate GALA (*F* = 38.01 df = 2; *P* < 0.001). There was no significant difference in progeny production between isolate VDTA and isolate GALA of *H. indica* (*P* > 0.05).

### Thermal tolerance

**Figure 8:**
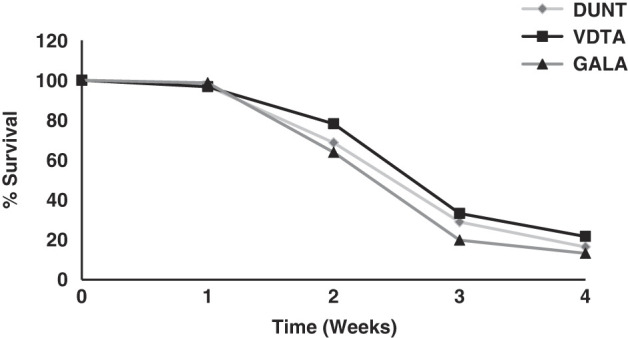
Mean percentage survival of IJ of isolates DUNT, VDTA and GALA following exposure at 40°C for 1, 2, 3, and 4 hr.

Survival of the tested nematode isolates decreased with time. Moderate survival was observed until 2 hr of exposure with 69%, 78%, and 64% of IJ of isolates DUNT, VDTA, and GALA surviving, respectively. After 4 hr, survival of isolate VDTA was highest (21.6%) compared to isolate DUNT (16.4%) and VDTA (13.2%) (Fig. 8). The survival of IJ after exposure at 40°C was significantly different for the tested isolates (*F* = 5.9; df = 2; *P* = 0.005). Exposure time to heat also had significant effect on IJ’s survival (*F* = 643.7; df = 3; *P* < 0.001). No mortality was observed in the control treatments. Isolate VDTA was significantly more tolerant than isolate GALA (*P* = 0.03). However, there was no significant difference in heat tolerance between isolate VDTA and isolate DUNT (*P* = 0.112).

### Infectivity at different soil moisture level

**Figure 9:**
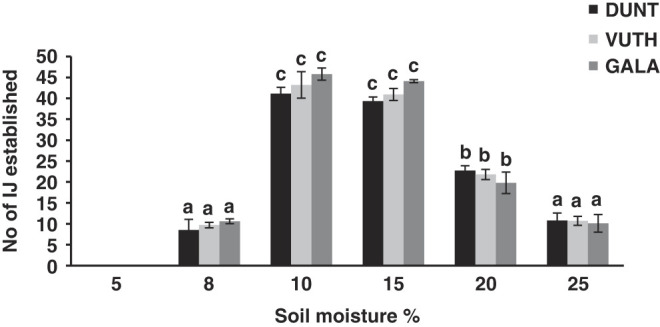
Effect of soil moisture (mean ± SE) on penetration of IJ of isolate DUNT, VDTA and GALA in *Galleria* larvae at moisture level 5%, 8%, 10%, 15%, 20%, and 25% (w/w). No IJ penetrated at moisture level 5%. Same lowercase letter above bars indicate no statistical differences among nematode isolates (*P* > 0.05).

All three native isolates of *H. indica* caused 100% mortality of *G. mellonella* larvae at all moisture levels except for 5% (Fig. 9). At 5%, all the tested isolates failed to cause any mortality of *G. mellonella*.

Their penetration rate increased until 10% of moisture level, and decreased with further increase in moisture level. Significant difference in number of nematodes penetrating into host at different moisture levels was detected for all the tested isolates (*F* = 827; df = 5; *P* < 0.001). However, there was no difference in penetration rate for moisture levels 8% and 25%. Similarly, there was no difference in penetration rate for moisture level 10% and 15% also. No significant differences in penetration rates were detected among the three tested isolates (*F* = 1.1; df = 2; *P* = 0.3) at different moisture levels.

### Rapid desiccation tolerance

**Figure 10:**
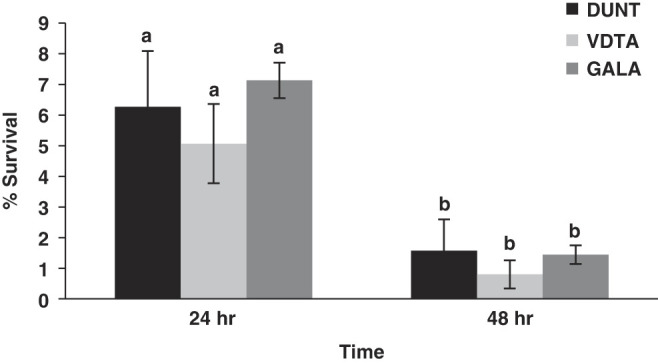
Percentage survival (Mean ± SE) of IJ of isolate DUNT, VDTA and GALA following exposure to 85% RH for 24 hr and 48 hr. Same lowercase letter above bars indicate no statistical differences among nematode isolates (*P* > 0.05).

Survival of IJ under 85% RH was affected by exposure time (*F* = 7.4, df = 1; *P* = 0.01), with 5.0 to 7.1% and 0.8 to 1.5% of IJ surviving on day 1 and 2 of the experiment, respectively (Fig. 10). The survival of IJ after desiccation did not differ significantly among the isolates (*F* = 0.2; df = 2; *P* = 0.7). Survival recorded in all control treatments during the 48 hr of the experiment was more than 95%.

### Hypoxia tolerance

**Figure 11:**
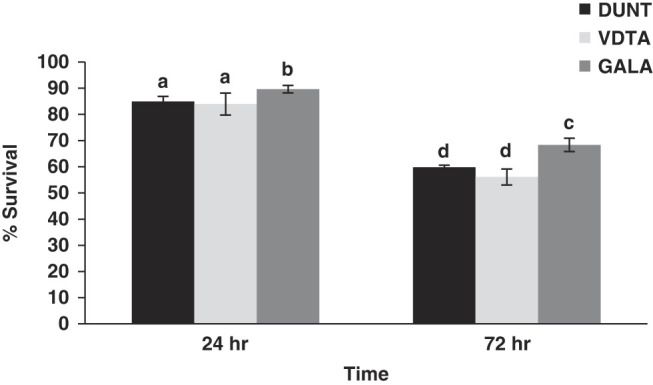
Percentage survival (Mean ± SE) of IJ of isolate DUNT, VDTA and GALA following hypoxic conditions for 24 hr and 72 hr. Same lowercase letter above bars indicate no statistical differences among nematode isolates (*P* > 0.05).

A gradual reduction of hypoxia tolerance, as indicated by IJ survival was observed during 72 hr exposure. The survival of IJ after hypoxia treatment differed significantly among isolates (*F* = 10.9; df = 2; *P* < 0.001) and exposure times (*F* = 225.7; df = 1; *P* < 0.001). Survival recorded in all control treatments during the 72 hr of the experiment was higher than 95%. After exposure to hypoxic conditions for 24 hr and 72 hr, survival of IJ varied between 83.9% and 89.6%, and 56.08% and 68.3%, respectively (Fig. 11). Above 80% the population of all tested isolates survived 24 hr of exposure to hypoxic conditions. After 72 hr, the highest survival was observed with isolate GALA (68.3%) thus showing the highest tolerance, which was significantly higher (Tukey test, *p* < 0.01) than in the other isolates tested.

### Survival capacity in storage

**Figure 12:**
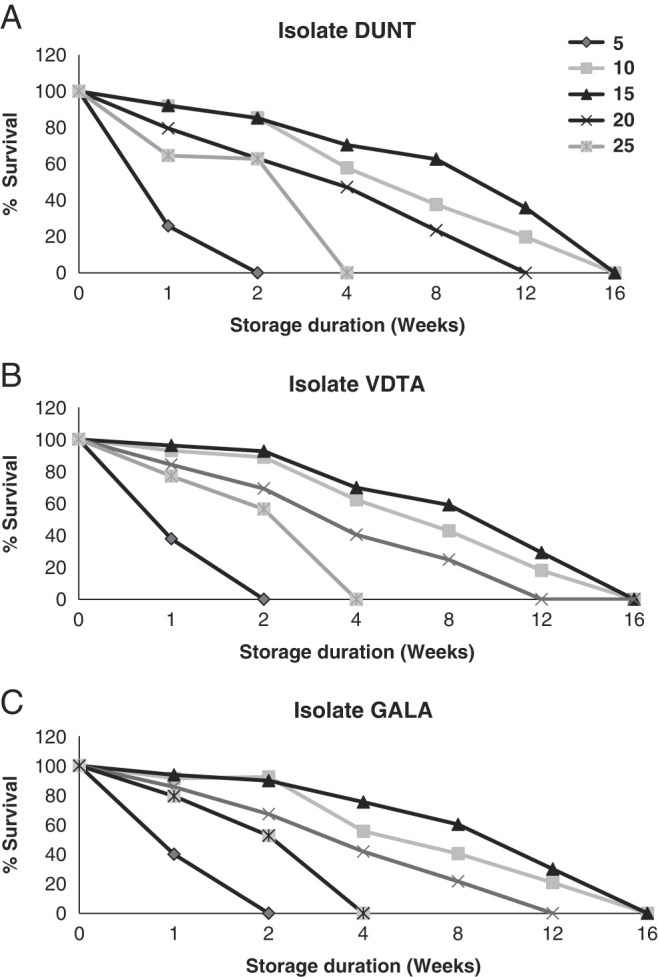
Survival capacity of isolates in storage stored at 5°C, 10°C, 15°C, 20°C, and 25°C for 1, 2, 4, 8, 12, and 16 weeks. (A) Effect of storage temperature and duration on % survival of the IJ of isolate DUNT. (B) Effect of storage temperature and duration on % survival of the IJ of isolate VDTA. (C) Effect of storage temperature and duration on % survival of the IJ of isolate GALA.

Storage capacity of the three isolates of *H. indica* was determined at temperatures ranging between 5°C and 25°C. There was a steady decline in the numbers of IJ alive in water and by week 16 of storage almost all IJ of *H. indica* were dead (Fig. 12). The optimal storage temperature for *H indica* in this study was observed around 15°C, followed by 10°C. The least survival of *H. indica* was observed at 5°C.

Survival of all the isolates was affected by storage temperature with significant differences among temperatures after 1, 2, 4, 8, 12, and 16 weeks of storage (*F* = 2546.1; df = 4; *P* < 0.001). There was a significant effect of duration of storage on survival (*F* = 2764.3; df = 5; *P* < 0.001). Significant differences in % survival was detected among three tested isolates (*F* = 14.2; df = 2; *P* < 0.001) with isolate GALA surviving at a higher rate than the other isolates (Tukey test, *p* < 0.05).

### Qualitative analysis of ecological traits among isolates

The ecological traits selected to identify the best candidate were reproductive capacity, heat tolerance, hypoxia tolerance and storage stability. Other traits were not considered as there was no significant difference among these isolates with respect to them. A maximum score of two points could be reached. However, all of the examined populations had moderate to low scores. The highest score was that of the isolate GALA (2 points) (Table 2), mainly due to its reproduction capacity and good score in environmental stress assays. The lowest score –3 was recorded for the isolate DUNT.

**Table 2.** Qualitative comparison of ecological traits among native isolates of *H. indica* DUNT, VUDT, AND GALA.

Isolate	Reproductive capacity	Heat tolerance	Hypoxia tolerance	Storage stability	Total
Isolate DUNT	-1	0	-1	-1	-3
Isolate VDTA	1	1	-1	-1	0
Isolate GALA	1	-1	1	1	2

## Discussion

Increasing knowledge about the ecological characteristics of a given EPN species isolated from different geographical locations contributes to an accurate estimation of the potential of these organisms as biological control agents (Del Valle et al., 2014). Ecological studies of EPN have shown clearly that not only species but isolates within a species of EPN also differ in their attributes such as host-finding ability, host range, infectivity, reproduction and environmental stress tolerance (Gaugler et al., 1989; Glazer, 2002; Hazir et al., 2001; Hominick, 1990; Morton and Garcia-del-Pino, 2009; Rolston et al., 2009; Rosa and Simoes, 2004; Shapiro and McCoy, 2000; Somasekhar et al., 2002). Populations of *H. indica* have also been reported to differ in their biological attributes and environmental tolerance (Yan et al., 2010). Distribution of *H. indica* throughout the tropical and subtropical region suggests that this species possesses a range of phenotypic characters which give it an advantage in this climatic zone. Such phenotypes might include adaptation to high temperatures, desiccation tolerance, and good dispersal ability (Stack et al., 2000). In current study, thirty-five native isolates of *H. indica* were screened for their virulence and the three most virulent isolates (GALA, VDTA, and DUNT) were selected for ecological characterisation.

In the present study, none of the isolates of *H. indica* showed nictating or vigorous jumping behaviour and showed strong directional response to volatile host cues confirming their cruiser behaviour. Even though, the IJ of native isolates of *H. indica* are longer (Kour et al., 2020) than other isolates of *H. indica* described in literature, their foraging behaviour is similar to them (Walia et al., 2008). However, this contradicts the finding of Susurluk et al. (2003) who reported different foraging behaviour for isolates of same *Heterorhabditis* spp. that differ in their length. In the present study, all isolates migrated over 10 cm, causing death of host within 48 hr in the soil column, demonstrating the high degree of cruising behaviour and dispersal abilities of the native isolates. In the vertical soil columns assay, establishment of IJ at different depths was similar to the establishment reported for active cruise forager with highest establishment occurring at 2 cm depth and the lowest occurring at 10 cm depth (Koppenhöfer et al., 2000). When compared to another cruiser *Steinernema glaseri* Steiner, 1929 (Rhabditida: Steinernematidae), the highest establishment rate is reported at depth of 5 cm (Koppenhöfer and Kaya, 1999). It is important to study the vertical movement of native *H. indica* IJ in order to be able to select suitable strain which can reach soil dwelling insect pests (Morton and Garcia-del-Pino, 2009). There are many economically important agricultural pests in Fiji, inhabiting soil and cryptic habitats that can be targeted using these native isolates.

In the present study, the dose-response test (LC50) values are similar to the values reported for *H. indica* by Noosidum et al. (2010). The thermal activity data obtained in this study also confirms that *H. indica* is more adapted to warm temperatures. The native isolates can infect *G. mellonella* in a range between 15°C and 35°C with optimum temperature of 25°C and conform to other reports on the optimum temperature of 25°C (Ebssa et al., 2004; Karunakar et al., 1999; Shamseldean et al., 1996). In exception is *H. indica* SAA2 strain from Egypt (Salem et al., 2008), whose temperature for infection ranges between 20°C and 35°C, with optimal pathogenicity recorded at 30°C. In the present study, the lowest temperature infectivity threshold for native isolates was recorded as 15°C below which they failed to induce larval mortality. However, Shapiro-Ilan et al. (2009) reported invasion and mortality in *G. mellonella* at 10°C by *H. indica* Hom1 strain. This indicates that thermal niche breadths for infection for Fijian isolates of *H. indica* is narrower than *H. indica* Hom1 strain but broader than for *H. indica* SAA2. Difference in thermal niche breadths for infection among strains of *Heterorhabditis megidis* (Poinar et al., 1987) (Rhabditida: Heterorhabditidae) has also been documented; the UK strain of *H. megidis* has a range of 5°C to 30°C vs 10°C to 30°C for the Dutch strain of *H. megidis* (Mason and Hominick, 1995). These results are in contrast to the statement made by Grewal et al. (1994) and observations made by Hazir et al. (2001). Grewal et al. (1994) stated that thermal niche breadths for infection differed among EPN species, but not among conspecific strains. He further stated that conspecific strains collected from different localities differed in infectivity at different temperatures, but not in their temperature activity ranges. Similar observations were reported by Hazir et al. (2001) demonstrating the conserved nature of the thermal niche breadth in the species of EPN. However, the results of current study and observation report by Mason and Hominick (1995) regarding temperature range and optimal temperature for infection for different species/populations of genus *Heterorhabditis* indicates that a fixed range and single optimal temperature cannot be assigned to its species. The thermal activity data further showed that penetration by IJ of native isolates of *H. indica* in host was highest at 25°C which is in close agreement with that reported by Karunakar et al. (1999).

The results of present study also showed that isolate VDTA is most thermal tolerant of the 3 native isolates tested. Isolate VDTA was obtained from a location that is characterised by high summer temperatures (daytime average > 30°C; Fiji Meteorological Service). This validates the correlation between heat tolerance of isolates and the mean annual temperature at the site of origin reported by Mukuka et al. (2010). The thermal tolerance of native isolates of *H. indica* is higher than the one reported for *H. indica* isolate from South Benin (1.9% survived at 40°C for 4 hr) (Zadji et al., 2014), China (strain CN 1- 50% survived at 40°C for 2 hr) and *H. indica* strain Hom1 (50% survived at 37°C for 3 hr) (Shapiro-Ilan et al., 2009) but lower than the one reported for the isolate of *H. indica* from West Bank (strains Beth 11 and Beth 22 – 80% survived 40°C for 3 hr) (Iraki et al., 2000) and China (strain ZZ68 – 95% survived 40°C for 2 hr) (Ma et al., 2013). Even though, Fijian isolates showed moderate thermal tolerances, it can be adjusted through laboratory acclimatisation involving prolonged storage or propagation at higher temperatures (Abu Hatab and Gaugler, 1997; Jagdale and Gordon, 1998), osmotic treatment (Feng et al., 2006; Yan et al., 2010) and heat-shock treatment (Selvan et al., 1996).

Reproduction thermal niche breadth of Fijian isolates was observed to be between temperature 20°C and 30°C, whereas at temperature 15°C and 35°C, progeny did not emerge from these cadavers. This could be due to the inability of symbiotic bacteria *Photorhabdus* spp. to multiply normally and provide nourishment to nematodes at extreme temperatures resulting in the reproductive failure of EPN at extreme temperature (Choo et al., 1998; Henneberry et al., 1996; Milstead, 1981). However, there is a strain 212-2 of *H. indica* that can reportedly reproduce at 35°C (Xu et al., 2010). The differences may be attributed to symbiont of *H. indica* strain 212-2 and needs further investigation. In the present study, the highest number of progeny production was observed at 25°C which is in close agreement with that reported by Karunakar et al. (1999). They observed highest number of emergence of *H. indica* IJ at 27.5°C. The highest number of emerged IJ was recorded for isolate VDTA (average 127,000 IJ/host) which is consistent with results for *H. indica* T2 (geographical origin: Thailand) (Maketon et al., 2011) but much less compared to the reproductive potential of *H. indica* Hom1 (300,000 IJ/host) (Nguyen et al., 2006). The differences may be attributed to differences in the climatic origins of these nematodes. Both, Fijian isolates and *H. indica* T2 are from tropical region and have similar reproductive potential which is less than the *H. indica* Hom1 which has been isolated elsewhere. Similar difference in reproductive potential of isolates of same species from different geographical origin has been reported by Hazir et al. (2001). They reported low reproductive capacity of the tropical isolate of *Steinernema feltiae* (Filipjev, 1934) (Rhabditida: Steinernematidae) MG-14 compared to Mediterranean isolates. The current study and observation reported by Hazir et al. (2001) highlights the possible effect of geographical origin on the reproductive potential of EPNs. Although the yield of native isolates in *G. mellonella* was less than that reported for *H. indica* Hom1, but other species that have similar reproductive potential have been successfully commercialized e.g. *Heterorhabditis bacteriophora* Poinar, 1975 (Rhabditida: Heterorhabditidae) and *S. feltiae* (Grewal et al., 1994; Nguyen et al., 2006). Significant differences in the reproductive potential of native isolates were recorded. Isolate DUNT had progeny significantly less than the isolate VDTA and isolate GALA. The number of penetrated IJ can also affect the multiplication potential of EPNs (Boff et al., 2000a; Costa et al., 2007). This is in agreement with the results of the penetration assay as lesser number of IJ of isolate DUNT were observed to have successfully established in the host compared to the two other isolates. The results also showed that emergence time of IJ decreased with increase in temperature and shortest emergence time of IJ was observed at 30°C. Boff et al. (2000b) observed that emergence time of IJ depends on initial number of IJ establishing in host larvae; IJ from cadavers exposed to a higher dose of IJ emerge earlier than those from cadavers exposed to a lower dose of IJ indicating that initial establishment of IJ in host larvae can affect emergence time. However, results of this study do not support this observation. In current study, maximum number of IJ established in host at 25°C as opposed to 30°C. Susurluk (2008) showed that the movement of IJ is temperature dependent. Early emergence of IJ of native isolates at higher temperature could be due to the high movement of IJ at 30°C which allows them to reach and establish within the host in less time.

For native isolates, the establishment of IJ inside the host started at all moisture levels except for 5%. This indicates that these isolates cannot persist in soil with low moisture level. The low potential of *Heterorhabditis* to survive low moisture level and desiccation has been discussed by Grewal et al. (2006), Mukuka et al. (2010), and Mejia-Torres and Saenz (2013). However, the result of this study is contrary to the results reported for *H. indica* strain T2 (geographical origin: Thailand) which has been reported to infect *G. mellonella* larvae at moisture level as low as 3% (Maketon et al., 2011), whereas Fijian isolates of *H. indica* were unable to cause any mortality at 5% moisture level. This suggests that the range of soil moisture at which native isolates of *H. indica* were effective is narrower than that *for H. indica* strain T2. The differences may be attributed to differences in the geographical location and/or habitat of these isolates. The native isolates were found either from riverside or from coastline locations and are more adapted to high moisture level. Another possible reason for this difference could be the size difference of IJ. Total body length of IJ isolated from Viti Levu (520-630 µm) is larger (Kour et al., 2020) then the body length reported for *H. indica* strain T2 (530µm). IJ needs a thin layer of water on the surface of soil particles for movement and infectivity (Negrisoli et al., 2013). For maximum movement, the layer of water should be as thick as nematode’s body diameter (Wallace, 1958). Koppenhöfer et al. (1995) speculated that the effect of soil moisture on nematode infectivity is correlated with nematode’s size. They hypothesized that small species can move through a thinner water film, whereas larger species require higher moisture levels. Since, *H. indica* strain T2 is smaller in size; it probably requires a thinner layer of water film than the larger isolates. In the field, low infectivity of native isolates at lower moisture level can be overcome by irrigating the field prior to the application. There are certain species of *Steinernema* that can infect the host in soil with moisture level as low as 1% (Gungor et al., 2006) and 2% moisture level (Kung, 1990). In the present study, the highest numbers of established IJ were observed at 10% and 15% moisture level which is in close agreement with reports for *H. indica* strain T2 (Maketon et al., 2011), *H. indica* isolate from Meghalaya, India (Yadav, 2012) and *H. bacteriophora* (Susurluk et al., 2001). Further increase in water content resulted in decline in number of invading IJ indicating that higher moisture level can have detrimental effects on the control potential of EPN (Koppenhöfer et al., 1995). Even though 100% mortality of host was observed at all moisture levels tested, there was a significant decrease in the number of established IJ with increasing moisture level. It is possible that when the soil is almost saturated, oxygen is less available and nematode movement is restricted (Simard et al., 2001). Species like *Steinernema anatoliense* (Hazir et al., 2003) (Rhabditida: Steinernematidae) ceases to infect the host at 20% moisture level (Gungor et al., 2006), whereas native isolates were able to infect and cause 100% mortality at 25% moisture.

The native isolates of *H. indica* showed very poor desiccation tolerance. These results are in agreement with the finding of O’Leary et al. (2001), Shapiro-Ilan et al. (2009), Ma et al. (2013), Shapiro-Ilan et al. (2014) and Zadji et al. (2014). Different strains of *H. indica* (Strain Ayogbe1, strain LN2, Strain HZG8, Strain KF-58 and Strain Hom 1) have been tested for desiccation tolerance and they all have shown poor desiccation tolerance. The possible reason for low tolerance of *H. indica* could be the non-adaptability to low moisture level. *H. indica* is a cruiser and can avoid low moisture level and desiccation by moving deep into the soil (Grewal et al., 2002). In general, low desiccation tolerance has been observed in *Heterorhabditis* species (Shapiro-Ilan et al., 2014; Surrey and Wharton, 1995). For formulation and long-term storage of native isolates of *H. indica*, attempts can be made to enhance their survival by pre-exposure to osmotic stress (Charwat et al., 2002) and warm storage at 35°C (Jagdale and Grewal, 2007).

In this study, native isolates showed good to moderate tolerance to anoxic conditions depending on the exposure time. The isolate GALA was most tolerant among all tested isolates as 68.3% of the total population survived after 72 hr exposure to hypoxic conditions. When compared, isolate GALA is more tolerant than *H. indica* strain Hom1 (20% and 5% survival after 24 hr and 72 hr, respectively) (Shapiro-Ilan et al., 2005) but similar to that of *H indica* strain Ayogbe1 isolated from Benin (85% and 60% survival after 24 hr and 72 hr, respectively). Studies of other *Heterorhabditis* species showed variability in survival, such as 0% survival in *H. bacteriophora* after 3 days of exposure (Shapiro-Ilan et al., 2005), 18% to 65% survival in *S. carpocapsae* Weiser, 1955 (Rhabditida: Steinernematidae) after 10 days of exposure (Somasekhar et al., 2002), 11.4% to 100% in *S*. *feltiae* after 1 day of exposure (Morton and Garcia-del-Pino, 2009) and 10% to 90% among populations of *H. bacteriophora* after 4 days of exposure (Grewal et al., 2002).

One of the major constraints on the commercialisation of heterorhabditids is the inferior storage stability and poor shelf life of formulated products (Elawad et al., 2001; Hiltpold, 2015; Strauch et al., 2004). In current study, even though the optimum concentration was 1000 IJ/ml for the storage experiment, poor storage stability was observed for native isolates of *H. indica* which is slightly higher when compared to Colombian *Heterorhabditis* sp. SL0708 (survive only up to 8 weeks) (Mejia-Torres and Saenz, 2013). Low longevity in *H. indica* population has also been reported by Shapiro-Ilan et al. (2006). During storage, dense aggregation of IJ of Fijian isolates were observed which could be because of their relatively big size (Kour et al., 2020). When stored in aqueous solution, *H. indica* has been reported to settle down and form precipitate on the bottom of the container (Askary et al., 2018). Agglomeration leaves the nematodes an environment of low oxygen content and loss of energy reserves which could be a factor for a shorter storage life of native isolate of *H. indica* (Andaló et al., 2010). A comparison between the different storage temperatures over time reveals that IJ of native isolates *H. indica* survive better when stored at 15°C, than at any other temperature. The same has been reported for *H. megidis* strain NLHE 87.3 which survives and performs better when stored at 10°C or 15°C, than at any other temperature (Boff et al., 2000b). When compared to Steinernema species, *Steinernema rarum* Doucet (1986) also has optimal storage temperature of 15°C but can survive much longer for 6 months (Koppenhöfer and Kaya, 1999). Native isolates also proved to be very susceptible to low temperature, as the highest mortality (100% after 15 days) was observed when stored at 5°C. In contrast, *H. indica* strain LN2 could survive for 24 days when stored at 5°C (Strauch et al., 2000). Warm adapted species are reported to perform poorly at lower temperature (5°C) (Elawad et al., 2001) demonstrating that there is a limit to cold temperature storage for warm adapted species. In the present study, intraspecific variation in survival of *H. indica* has been observed for three native isolates. Intraspecific variation in survival has been reported for other *Heterorhabditis* species as well. Fitters and Griffin (2006) studied the survival of *H. megidis* isolates in water at 20°C and concluded that intraspecific variation in survival is due to variation in rates of depletion of energy reserves, which in turn is associated with levels of locomotor activity.

For the qualitative analysis, only reproductive potential, heat tolerance, hypoxia tolerance and storage stability of isolates were compared. Other traits were not considered as there was no significant difference among these isolates with respect to them. The results obtained in the analysis identified isolate GALA as superior isolate, even though it performed poorly in desiccation tolerance test. Qualitative comparison of ecological traits among different EPN species or strains of a single species has been done by many researchers (Morton and Garcia-del-Pino, 2009; Salame et al., 2010; Shapiro-Ilan et al., 2003). These researchers compared the ecological traits of different EPN species (Morton and Garcia-del-Pino, 2009) or strains of a single species (Shapiro-Ilan et al., 2003) for control of one particular pest. Salame et al. (2010) used this method to compare newly isolated populations of EPN during survey of Israel and recommended its use for the general characterisation and comparison of new EPN populations isolated during other surveys (Salame et al., 2010). Some researchers have used Hierarchical Cluster Analysis to cluster the isolates that scored close on the traits tested and to select superior EPN isolates (Campos-Herrera et al., 2007; Campos-Herrera et al., 2008; Ma et al., 2013; Rosa and Simoes, 2004; Zadji et al., 2014). However, Ma et al. (2013) reported the failure of hierarchical approach in distinguishing isolates. According to them, this method can place poor and superior isolate in one cluster. They suggested conducting both methods of analysis and weigh the two together when deciding which strains to carry forward for biological control.

## Conclusion

In the present study, newly isolated populations of *H. indica* were compared in a series of bioassays designed to evaluate key traits that can make them an effective biological control agent. The temperature data showed that native isolates of *H. indica* are warm-adapted nematodes with thermal range for infectivity between 15˚C and 35˚C and can reproduce between 20˚C and 30˚C. They are highly virulent with LC50 values against *G. mellonella* ranging from 2.8 to 3.8 IJ/larva. However, they showed poor desiccation tolerance and fail to infect host in soil with moisture level below 8%. They showed moderate level of hypoxic tolerance and can be stored at 15˚C for 4 months. The comparison of ecological traits identified the isolate GALA as a superior isolate. The results of this experimental study on ecological aspects of these native isolates of *H. indica* should form a basis for their potential use in biological control of insect pests in Fiji.
